# A forecasting tool for a hospital to plan inbound transfers of COVID-19 patients from other regions

**DOI:** 10.1186/s12889-024-18038-3

**Published:** 2024-02-16

**Authors:** Mehmet A. Begen, Felipe F. Rodrigues, Tim Rice, Gregory S. Zaric

**Affiliations:** 1 Ivey Business School and Western University, London, Canada; 2 Department of Epidemiology and Biostatistics, Western University, London, Canada; 3 Department of Statistical and Actuarial Sciences, Western University, London, Canada; 4 King’s University College at Western University, London, Canada; 5 London Health Sciences Centre, London, Canada

**Keywords:** COVID-19, Simulation, Spreadsheet, Capacity planning, Emergency response

## Abstract

**Background:**

In April 2021, the province of Ontario, Canada, was at the peak of its third wave of the COVID-19 pandemic. Intensive Care Unit (ICU) capacity in the Toronto metropolitan area was insufficient to handle local COVID patients. As a result, some patients from the Toronto metropolitan area were transferred to other regions.

**Methods:**

A spreadsheet-based Monte Carlo simulation tool was built to help a large tertiary hospital plan and make informed decisions about the number of transfer patients it could accept from other hospitals. The model was implemented in Microsoft Excel to enable it to be widely distributed and easily used. The model estimates the probability that each ward will be overcapacity and percentiles of utilization daily for a one-week planning horizon.

**Results:**

The model was used from May 2021 to February 2022 to support decisions about the ability to accept transfers from other hospitals. The model was also used to ensure adequate inpatient bed capacity and human resources in response to various COVID-related scenarios, such as changes in hospital admission rates, managing the impact of intra-hospital outbreaks and balancing the COVID response with planned hospital activity.

**Conclusions:**

Coordination between hospitals was necessary due to the high stress on the health care system. A simple planning tool can help to understand the impact of patient transfers on capacity utilization and improve the confidence of hospital leaders when making transfer decisions. The model was also helpful in investigating other operational scenarios and may be helpful when preparing for future outbreaks or public health emergencies.

**Supplementary Information:**

The online version contains supplementary material available at 10.1186/s12889-024-18038-3.

## Background

In April 2021, the province of Ontario, Canada, was at the peak of the third wave of COVID-19 cases [1, 2]. The highest daily number of new infections during the third wave, 5067, occurred on April 14, 2021 [2]. Cases were highest in the Toronto metropolitan area, the largest urban area in the province, and many ICUs in the Toronto area were full. As a result, some COVID patients from the Toronto metropolitan area who required hospital care had to be transferred to other regions of the province [3, 4].

The London Health Sciences Centre (LHSC) in London, Ontario, is an academic, tertiary care hospital with two sites, each with a medicine ward and ICU. In April 2021, requests were made to transfer COVID patients to LHSC from other regions. The requests created a challenge for leaders at LHSC: accepting patients from other regions would increase bed utilization of LHSC facilities and thus potentially reduce the ability of LHSC to care for patients from within their normal catchment area. However, denying the request could have serious health consequences for patients needing transfer and hospitals requesting the transfer which were already short on capacity.

To help leaders at LHSC make decisions about accepting regional transfers, we developed a spreadsheet-based tool to help forecast the utilization of their ICUs and medicine wards up to seven days into the future. The tool uses knowledge of current admission rates, length-of-stay, and assumptions about future arrivals of patients from outside the region to make short-term census projections.

## Methods

### Model overview

**Fig. 1:**
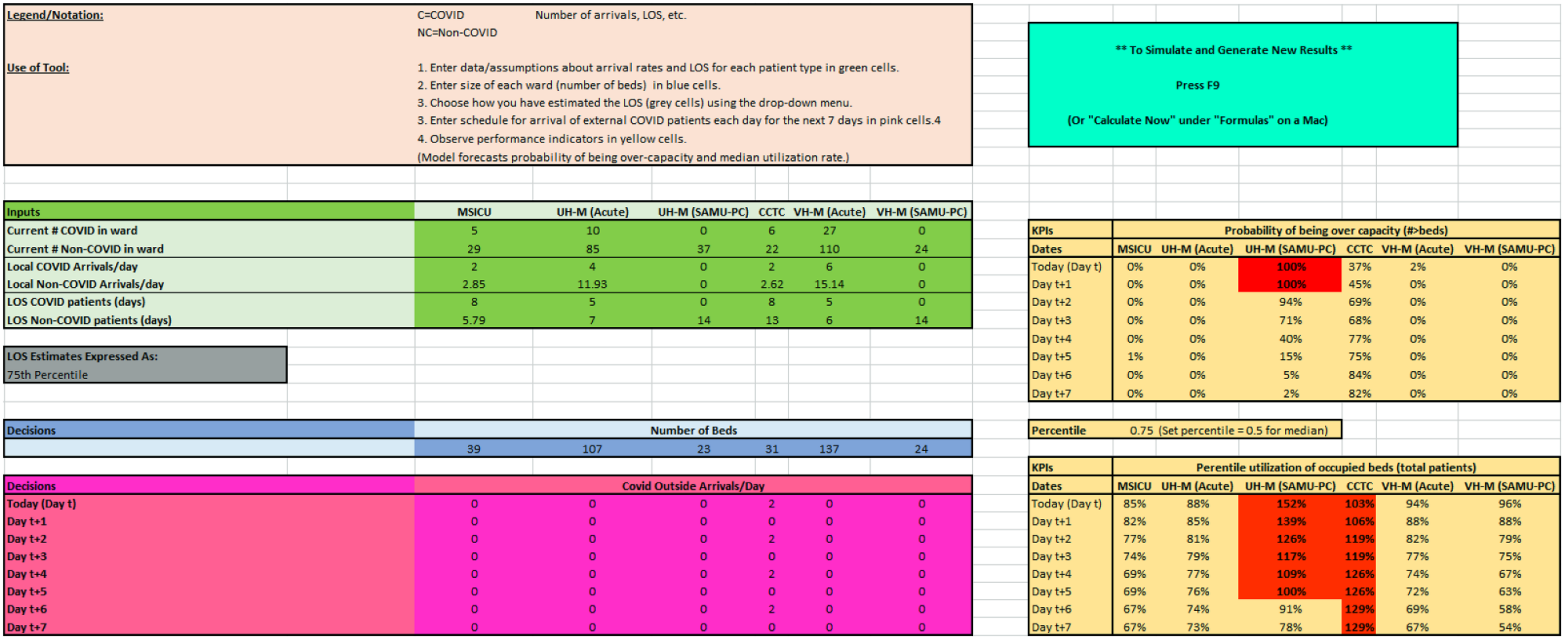
Screen capture of the input section of the model ^†^. ^†^A user enters three different types of information into the tool: (1) Inputs (green): Current number of (COVID and non-COVID) patients, arrivals for all local patients, LOS for all patients. (2) Decisions (blue): Number of beds to operate. (3) Decisions (pink): Non-local COVID arrivals, i.e., COVID patients, are to be accepted from other jurisdictions. Then the model calculates two tables (yellow): The probability of over-capacity and percentile utilization of occupied beds. (The user can choose the percentile level, e.g., 50% for median utilization). We see these in the yellow tables on the right, and the model highlights in red days when estimated capacity utilization exceeds 100%

We developed a spreadsheet-based Monte Carlo simulation model [5] to investigate the impact of accepting transfer patients from other hospitals on ward capacity levels at LHSC. The model focuses on capacity in six wards: ICU (referred to in the tool as Medical-Surgical Intensive Care Unit (MSICU) at the University Hospital site and the Critical Care Trauma Centre (CCTC) at the Victoria Hospital (VH) site), as well as Acute and Sub-Acute Medicine (SAMU), at each of two LHSC sites. The impact of patient transfers is characterized by bed utilization rates and probabilities of exceeding hospital capacity in each of the six wards. The model was developed in Microsoft Excel (Fig. 1) to facilitate rapid development and deployment and ease of use by staff at LHSC.

### User input– hospital data

Data is entered into three portions of the model. The first set of data inputs relates to the current state of the six hospital wards and their recent patient volumes (the green cells in Fig. 1). A model user enters the number of COVID patients (“COVID patients”) and patients with other conditions (“non-COVID”) that are currently in each of the six wards; the arrival rate of new COVID and non-COVID patients to each of the six wards, expressed as the average number of patients per day; and a number representing the length-of-stay (LOS) for the two types of patients in each of the six wards. The LOS values are intended to represent the total amount of time that each type of patient would occupy a bed in each ward and thus account for any cause of disposition from the ward, including discharge, transfer to another ward or hospital, or death. All LOS values are assumed to be exponentially distributed. To fit the exponential distribution, the user enters additional information using a drop-down menu. In particular, the user specifies whether the LOS value represents the LOS distribution’s mean, median, 75th percentile, or 90th percentile (gray cells in Fig. 1). The LOS information is used to simulate the number of patients of each type who leave each ward per day, as described in the Simulation Model section.

The second set of inputs required is the number of beds in each ward (blue cells in Fig. 1). Changing these parameters meant the hospital could open additional beds, create off-site capacity in a field hospital setting, or reallocate beds from different wards, such as cancelled elective surgery beds. The third input specified by the model user is the number of planned transfers of COVID patients from outside the catchment area for the current day and the next seven days into the future (pink cells in Fig. 1).

### Simulation model

**Fig. 2:**
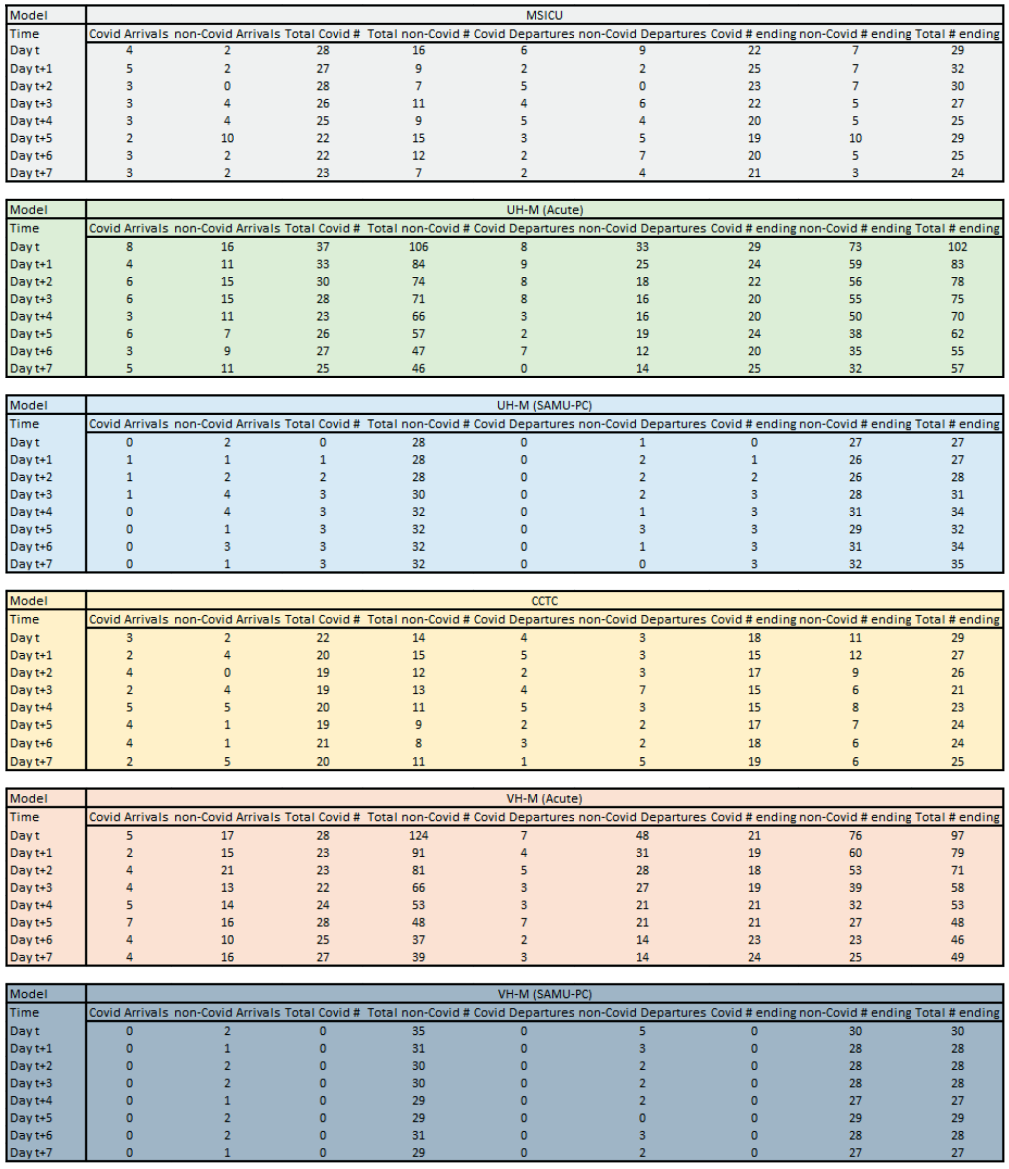
Patient flows at wards*. *The model keeps track of arrivals (COVID and non-COVID), all departures and the number of patients who end up staying at each ward and each day. The model treats the current day as ‘t’ and calculates the mentioned quantities for the next seven days (t + 1 to t + 7)

For each of the six simulated wards, the total patient counts each day for the next seven days were calculated using the following steps (Fig. 2). The day of planning is denoted by day t; subsequent days in the planning horizon are days t + 1, t + 2,…, t + 7. Full details are described in the appendix.

1. Simulate the number of new COVID and non-COVID patients in each ward daily. For COVID patients, the number of new arrivals is the sum of the planned intake from other regions and the number of new arrivals from the local region.
2. Simulate the number of patients of each type to leave each ward each day. As noted earlier, we assumed that LOS was exponentially distributed. Based on user-specified information, we estimated the parameter for each exponential distribution, then used that to calculate the probability that a patient exits the ward each day. The total number of patients who exit the ward is simulated from a binomial random variable where the number of trials (n) is the sum of the number of patients in the ward at the beginning of the day and the number of arrivals that day, and the probability of success (p) is the probability that a patient exits the ward.
3. Update the census in each ward at the end of each day. The number of each patient type at the end of each day is the number of patients at the end of the previous day plus the number of patients who arrive minus the number of patients who exit.
4. Steps 1–3 are repeated to generate an end-of-day census up to the end of the 8th day into the future for each of the six wards.

Note that all variability in the model is a result of steps 1 (variability in the daily number of arrivals) and 2 (variability in the daily number of exits from each ward). Since steps 1 and 2 involve random variables, the model is run hundreds of times to generate a distribution of possible outcomes. For this application, the model was run 400 times. This size was chosen to balance the need to produce a wide range of outcomes with the ability for the model to execute quickly on a wide range of computers. In preliminary testing of the model, we found that model results were fairly stable beyond 200 runs. Note that the model was constructed to enable an end user to change the number of runs if it was later determined that more runs were needed.

### Model outputs

The model has two types of outputs (yellow cells in Fig. 1).

1. The probability that each of the six wards will be over capacity at the end of each day for the planning horizon.
2. Ward utilization for a target percentile (from 0 to 100) of the simulation runs. For example, if the target percentile is 75 and the corresponding ward utilization value is 87%, the 75th percentile of simulations had 87% ward utilization.

For both outputs, conditional formatting was used to highlight in red those days when estimated utilization would exceed ward capacity.

### Model use

The model was intended to be used frequently on a “rolling horizon” basis [6], meaning that the plan is re-evaluated as new information becomes available.

## Results

### Preparation and response from end users

Given the impact of the third wave of the pandemic, the hospital recognized the need for an objective tool to support short-term capacity planning decision-making. This was necessitated by the need to balance an increasing volume of patients admitted for COVID related illness while also maintaining as much scheduled service as possible. This was particularly important for understanding the impact to planned surgeries. It was important to have a model that could be developed and deployed quickly to help guide decisions and planning. The tool described in this paper was operationalized in May 2021 and used once or twice per week, as needed, until February 2022.

**Fig. 3:**
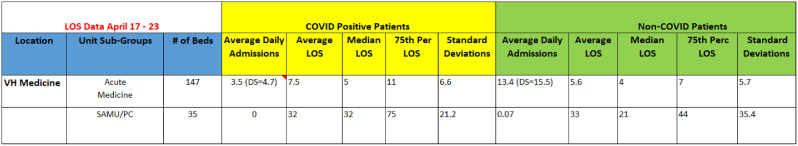
Screen capture of input data **. **Input data captures the arrival rate of new COVID and non-COVID patients to each of the six wards and the length-of-stay (LOS) for the two types of patients in each of the six wards based on a seven-day retrospective data collection. This example captures the data for the acute and sub-acute medicine wards at Victoria Hospital

Model input data was collected from several sources. The number of COVID patients and patients with other conditions occupying beds in each of the six wards was captured using the hospital’s daily occupancy report. The hospital’s Decision Support team provided the previous week’s arrival rate of new COVID and non-COVID patients and the length of stay for these patients (Fig. 3). The number of beds in operation was based on the bed census at the time of simulation (blue cells in each scenario). The expected number of COVID patient transfers from outside LHSC’s catchment area, “COVID Outside Arrivals/day,” was directed by the regional and provincial COVID Command Centre.

The model outputs, including the probability that each ward would exceed bed capacity and the projected impacts on occupancy, were reported at least once per week to the hospital’s COVID Operations Executive Leadership team and more frequently when the pandemic warranted a nimbler response. Specific projection scenarios, such as the impact of significant changes in hospital admission rates, were also requested by the hospital’s executive team and were used to inform the hospital’s response.

Reporting to the hospital’s COVID Operations Executive Committee, the hospital’s COVID Operations Director lead would present the current state impact, as well as multiple scenarios derived from the simulation tool to inform the committee’s decision making. Scenarios presented included the need to open new ward and ICU beds as well as reducing planned patient activity to ensure enough ward and ICU bed capacity to meet future demand.

Before using the model, the Chief of Critical Care at LHSC, together with the director lead, offered a virtual session with physicians and hospital leaders that were members of the COVID Operations Executive Committee to explain the model logic and build confidence in the tool. The tool was met with enthusiasm given its ability to objectively leverage real time data in the wards most heavily impacted by COVID patient volumes to make the most informed decisions possible. The tool was subsequently used over a ten-month period during perhaps the most challenging time during the COVID pandemic.

### Incorporation into regular planning at LHSC

In most scenarios, the estimates that the tool projected were reliably matched with the system occupancy that was realized in the subsequent seven days. Because of its conservative design, there were times when bed capacity in each of the wards was more than sufficient to meet the COVID specific patient demands and as a result planned activity was expanded. While the original intended use of the tool was to inform the number of transfers that could be accepted, the utility of the tool expanded to include the ability to measure the impact of bed closures due to intra-hospital COVID outbreaks, to balance the COVID response with planned hospital activity, (e.g. surgeries) and to inform the human health resource requirements to maintain staffing levels in the wards. The hospital also considered how a similar tool might help inform future bed allocation decisions in everyday operations.

### Scenarios

We illustrate the model with three representative scenarios that build on each other. Scenario 1 represents a case with high baseline utilization across all six wards, with COVID-positive patients occupying 11 − 21% of the ICU and acute medicine beds and no external COVID patient arrivals. Scenario 2 represents the impact of adding 20 acute medicine beds at UH and ten additional acute medicine beds at VH. Scenario 3 represents the potential impact of accepting two additional ICU patients to the VH site every other day. The model user enters arrival rates and LOS values based on retrospective data from the previous seven days.

**Fig. 4a:**
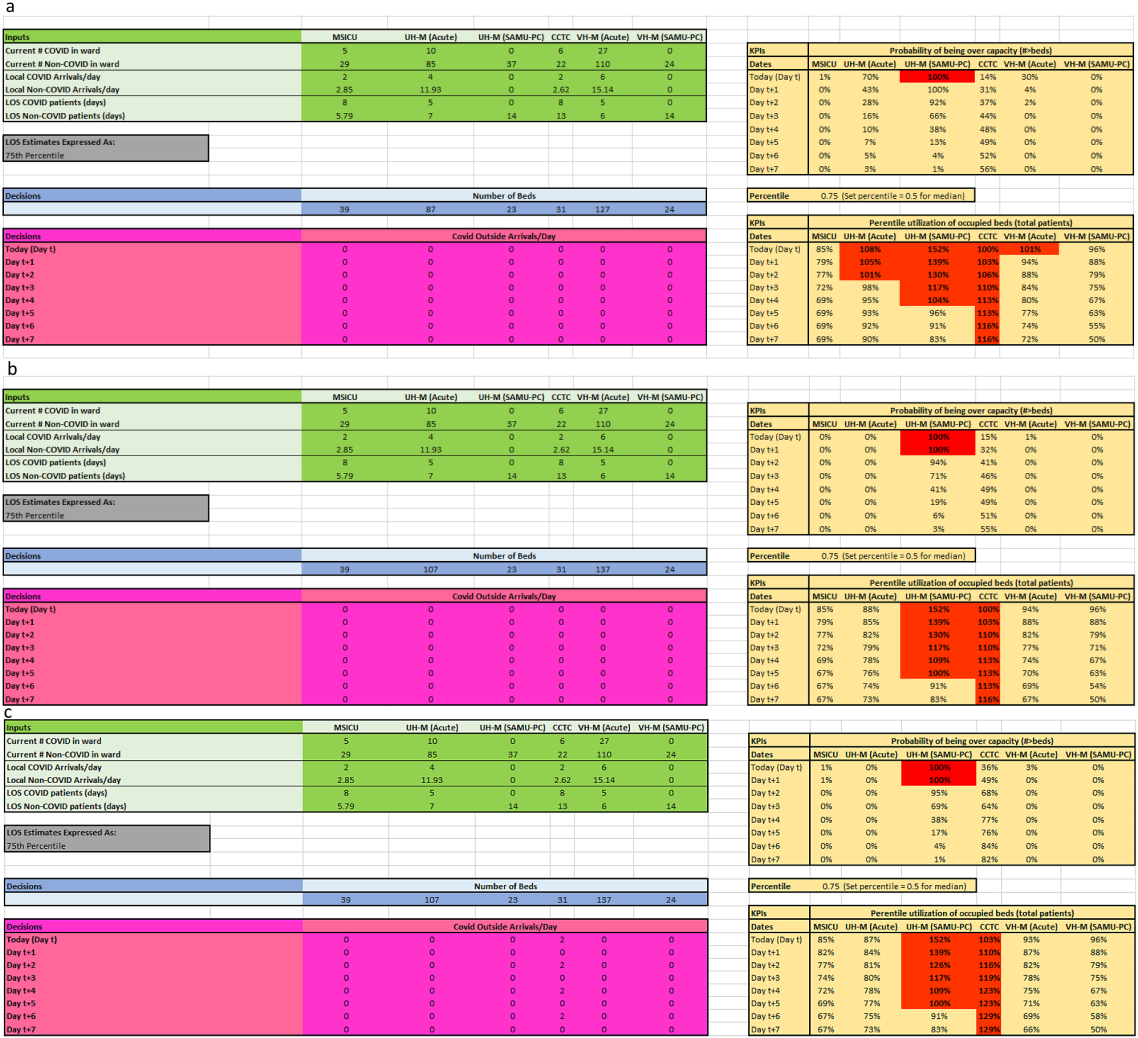
Screen captures of different scenarios. (**a**) Scenario 1. (**b**) Scenario 2. (**c**) Scenario 3

In scenario 1 (Fig. 4a), the combination of high utilization across all six wards and the impact of COVID-positive patient volumes result in the acute medicine wards at both sites exceeding 100% utilization and needing additional beds to support patient volumes. In addition, the model demonstrates the need for the ICU at the VH (CTCC) site to load level with the ICU at the UH (MSICU) site.

Scenario 2 (Fig. 4b) depicts the identical bed pressures of scenario 1. In addition, 20 additional beds are added to acute medicine at UH, and ten additional beds are added to acute medicine at VH. The utilization rate in the acute medicine wards decreases below 100% at both sites.

In scenario 3 (Fig. 4c), all scenario inputs from scenario 2 are maintained and only the outside COVID patient arrivals at VH ICU change (pink cells) with the consideration to accept two additional patients every other day over seven days. The model demonstrates that if the patients are to be accepted then the need to either load level or add additional beds at the VH ICU becomes critical as the ICU bed utilization at VH exceeds 120% by day t + 4.

Over time, the use of the model evolved, and the model was used to evaluate new scenarios. One scenario was the reallocation of bed designations within the hospital, such as changing the designation of surgery ward beds to medicine ward beds. A second scenario was the modeling of outbreaks at one site, which would force all admissions to be directed to the other site.

## Discussion

In this paper, we described a tool developed to help a hospital plan for transfers of COVID patients from other regions during a surge in cases. The model was incorporated into the planning process starting in May 2021. Although initially developed to manage transfers within the province, a model like this may have benefits in helping hospitals manage transfers over wider geographic regions. For example, in October 2021, LHSC received transferred COVID patients from Saskatchewan when that province had high case counts [7]. As noted, the model was used over a 10-month period, from May 2021 to February 2022.

Despite being built for LHSC, the method is easily generalized. A hospital could relabel the column headings in this model to tailor the wards to their situation. A hospital having more than six wards could add additional wards to the spreadsheet.

Other research teams developed methods to make decisions about admitting individual patients to an ICU [8]. These methods recognize the trade-offs involved in making this decision: “ICU admission in this situation may also deprive another patient who has a chance of recovery in the event of ICU admission from receiving ICU care [8].” However, they do not explicitly model the impact of admission decisions on bed capacity.

We identified a small number of papers in which spreadsheets were used for COVID management applications [9–11] but none that were used to forecast future occupancy or help in planning patient transfers. Spreadsheet simulation models have also been developed to support decision making and preparedness in other domains, including pandemic influenza preparedness [12], physician workforce planning [13], kidney exchanges [14], foot-and-mouth disease [15], and emergency department flow [16].

Other tools with similar goals have been developed. For example, Baas et al. developed a tool to plan regional ICU transfers in the Netherlands [17]. Dijkstra et al. developed a model to create a “fair balancing” of hospital COVID patients [18]. Castro et al. developed a tool to forecast demand for hospital beds, ICU beds, and ventilators in New Mexico [19]. Donker et al. developed a model to predict demand at a tertiary care center in Germany [20]. Ortiz-Barrios et al. developed a discrete-event simulation model. They illustrated the performance of the model with data from a hospital in Spain [21]. Several tools with similar objectives have also been developed in other settings (see [22–24] for reviews).

Like any model, our tool has limitations. We assumed that the wards were independent. That may not be true, as patients don’t always flow downstream, and may require multiple ICU stays per hospital admission. We circumvented the dependence between wards by incorporating internal patient movements into the arrival rates to each ward. Also, a rolling horizon planning approach with frequent updates allows ward capacities to be updated frequently– before they would likely cause significant problems. The number of runs for the model was initially selected without a formal sample size analysis. However, the model was built in a way that would easily allow the end user to change the number of runs if they determined that a larger run size was needed. We assumed that ward LOS for all patients followed an exponential distribution, allowing us to exploit its “memoryless property” [5]. We used the exponential distribution to calculate the daily probability of ward discharge for any patient, then used this probability as a parameter in a binomial distribution to calculate the number of ward exits per day. Using another distribution that does not have the memoryless property (e.g. Weibull) would make it challenging to model departures in a spreadsheet because departures on any given day would not be constant, but would depend on the history of previous arrivals, including the history of arrivals before the simulated time window. More sophisticated whole-hospital simulation models exist (e.g., [25]). However, these often require specialized software, limiting the ability to distribute them widely, and they typically require extensive time and effort to develop and validate.

## Conclusions

This work shows that a relatively simple model can be developed quickly and benefit end users. This was emphasized in a letter written by the interim President and CEO of LHSC, who wrote, “…we now have a more reliable way to predict the impact of receiving patients from hospital partners on top of local unscheduled care needs. The modelling is flexible and gives us the confidence we were looking for to ensure we can continue to safely serve our own community while helping ease the pressures in harder hit areas.” The immediate need for this model at LHSC has passed. However, understanding how to quickly develop and implement a simple model like this, in partnership with hospital and public health decision-makers, can be helpful when planning for future public health emergencies [26].

### Electronic supplementary material

Below is the link to the electronic supplementary material.

Supplementary Material 1: Simulation Details

Supplementary Material 2: Spreadsheet Model

## Data Availability

The spreadsheet file described in this manuscript has been included as a supplementary file.

## References

[1] Government of Ontario. Case numbers, spread and deaths. 2022 [cited 2022 March 22 2022]; Ontario government covid reporting page]. Available from: https://covid-19.ontario.ca/data/case-numbers-and-spread.

[2] Public Health Ontario. Ontario COVID-19 data tool. 2022 [cited 2023 May 17 2023]; Available from: https://www.publichealthontario.ca/en/data-and-analysis/infectious-disease/covid-19-data-surveillance/covid-19-data-tool?tab=trends.

[3] Bieman J. London hospital’s COVID patient count hits new high as area cases ease, in London Free Press. 2021: London, ON.

[4] Bieman J. Toronto COVID crisis sending patients to London-area’s smallest hospitals, in London Free Press. 2021: London, ON.

[5] Hillier FSL, Gerald J. Introduction to operations research. 2015, New York, NY: McGraw-Hill. 1010.

[6] Sethi S, Sorger G (1991). A theory of rolling horizon decision making. Ann Oper Res.

[7] Bieman J. London hospital taking in patients as COVID fourth wave hits Saskatchewan. 2021 [cited 2021 November 1, 2021]; Available from: https://lfpress.com/news/local-news/london-hospital-taking-in-patients-as-covid-fourth-wave-hits-saskatchewan.

[8] Azoulay E et al. Admission decisions to intensive care units in the context of the major COVID-19 outbreak: local guidance from the COVID-19 Paris-region area. Crit Care, 2020. 24(1). 10.1186/s13054-020-03021-2 PMC7274070 32503593

[9] Joventino WDD, dos Santos LKG, Roque ACM (2023). To present the form of organization of the information related to the notifications of suspected and/or confirmed cases of COVID19 to better support the decision making process in the pandemic in the municipality of Jucurutu/RN. Revista De Gestao E Secretariado-Gesec.

[10] Meyer J, Lima M (2023). Relevant mathematical modelling efforts for understanding COVID-19 dynamics: an educational challenge. Zdm-Mathematics Educ.

[11] Salem A et al. Improving management of hospitalised patients with COVID-19: algorithms and tools for implementation and measurement. Bmj Open Qual, 2020. 9(4). 10.1136/bmjoq-2020-001130 PMC7670554 33199287

[12] Stein ML (2012). Development of a resource modelling tool to support decision makers in pandemic influenza preparedness: the AsiaFluCap Simulator. BMC Public Health.

[13] Relić D, Fišter K, Božikov J (2019). Using Simulation modeling to inform policy makers for planning physician workforce in Healthcare System in Croatia. Stud Health Technol Inf.

[14] Karademirci O (2015). Implementation of a User-Friendly, flexible Expert System for selecting optimal set of kidney exchange combinations of patients in a transplantation Center. Transpl Proc.

[15] Ap Dewi I, Molina-Flores B, Edwards-Jones G (2004). A generic spreadsheet model of a disease epidemic with application to the first 100 days of the 2001 outbreak of foot-and-mouth disease in the UK. Vet J.

[16] Klein MG, Reinhardt G (2012). Emergency department patient flow simulations using spreadsheets. Simul Healthc.

[17] Baas S (2021). Real-time forecasting of COVID-19 bed occupancy in wards and intensive care units. Health Care Manag Sci.

[18] Dijkstra S (2023). Dynamic fair balancing of COVID-19 patients over hospitals based on forecasts of bed occupancy. Omega.

[19] Castro LA (2021). How New Mexico leveraged a COVID-19 case forecasting model to preemptively address the Health Care needs of the state: quantitative analysis. JMIR Public Health Surveill.

[20] Donker T (2021). Navigating hospitals safely through the COVID-19 epidemic tide: Predicting case load for adjusting bed capacity. Infect Control Hosp Epidemiol.

[21] Ortiz-Barrios M et al. Artificial intelligence and discrete-event simulation for capacity management of intensive care units during the Covid-19 pandemic: a case study. J Bus Res, 2023. 160. 10.1016/j.jbusres.2023.113806 PMC9981538 36895308

[22] Smith-Daniels VL, Schweikhart SB, Smith-Daniels DE (1988). Capacity Management in Health Care Services: review and future research Directions*. Decis Sci.

[23] Bhattacharjee P, Ray PK (2014). Patient flow modelling and performance analysis of healthcare delivery processes in hospitals: a review and reflections. Comput Ind Eng.

[24] He L (2019). A systematic review of research design and modeling techniques in inpatient bed management. Comput Ind Eng.

[25] Rodrigues FF, Zaric GS, Stanford DA (2018). Discrete event simulation model for planning level 2 step-down bed needs using NEMS. Oper Res Health Care.

[26] Walensky RP, What I. Need to Tell America Before I Leave the C.D.C., in The New York Times. 2023: New York.

