# Supplementary material for: A forecasting tool for a hospital to plan inbound transfers of COVID-19 patients from other regions
Source: BMC Public Health. 2024 Feb 16;24:505. doi: 10.1186/s12889-024-18038-3 (PMC10874054; doi:10.1186/s12889-024-18038-3)
Supplement: Supplementary file 1 — Supplementary Material 1: Simulation Details [file 12889_2024_18038_MOESM1_ESM.docx]

**Supplemental Material:**

**A Forecasting Tool for a Hospital to Plan Inbound Transfers**

**of COVID-19 Patients from Other Regions**

Mehmet A. Begen, Felipe F. Rodrigues, Tim Rice, Gregory S. Zaric

Monte Carlo Simulation in Microsoft Excel

Monte Carlo simulation is a computer technique that is used to generate a range of possible outcomes for a system that is subject to uncertainty. Uncertain quantities are sampled from appropriate probability distributions, where the probability distributions represent the distributions of known input values to the system. One iteration of a simulation model represents a single possible outcome for the system. Monte Carlo simulation models are usually run hundreds or thousands of times to generate a range of possible outcomes. After the simulation has been run, statistics are generated for important outcome measures (e.g., mean, variance, percentiles of distributions). A common use for simulation models is to systematically vary control parameters and investigate how changes in control parameters affect output statistics.

Monte Carlo simulation can be accomplished in Microsoft Excel using several built-in functions [1]. In particular,

- RAND() generates a realization from a random variable that is uniformly distributed between 0 and 1. Every instance of the function RAND() will generate a new value between 0 and 1 every time the spreadsheet recalculates.
- NORM.INV(RAND(), mean, standard deviation) generates a realization from a normal random variable with the specified mean and standard deviation.
- BINOM.INV(number trials, probability, RAND()) generates a realization from a binomial random variable with the given number of trials and probability of success.

A spreadsheet model using these formulas will generate a single instance of the simulated system. Multiple instances can be simulated using the Data Tables feature or VBA programming.

Simulating the Number of Daily Arrivals

We approximate the number of arrivals using a normal distribution and call this the “Raw number of arrivals.” We truncate at zero to avoid negative values and round to whole numbers yielding the actual simulated number of arrivals. Thus, the daily number of arrivals is given by:

Raw number of arrivals = NORM.INV(RAND(),average # arrivals, SQRT(average # arrivals))

Actual number of arrivals = ROUND(max(0, Raw number of arrivals))

The initial assumption that the mean and standard deviation of the distribution for the number of arrivals would be equal was based on the common assumption in queuing and discrete event simulation models that arrivals follow a Poisson process, where the mean and standard deviation of the arrival distribution are the same [2, 3]. Note that the user can modify the formula for the number of arrivals if local experience suggests that the standard deviation should differ from the abovementioned amount.

## Fitting an Exponential Length-of-Stay (LOS) Distribution

We modelled LOS using an exponential distribution. The exponential distribution has the “memoryless property,” [2] meaning that the distribution of the remaining LOS for a patient does not depend on the time they have already spent in the ward. This property implies that we do not need to know when each patient arrives in a ward; we only need to know how many patients are in a ward daily. Without this property, it would be challenging to build a model like this in a spreadsheet, as we would need to track each patient and update their probability of discharge each day based on the number of days spent in the ward so far. It would also increase the complexity of the information a model user requires as they would need estimates of ward census for each of the six wards as well as the day of arrival for *every* patient.

The exponential distribution has a single parameter, typically represented using the notation λ, where 1/λ is equal to the mean of the distribution. In our model, the user must enter a number representing information about patient LOS and indicate what that number represents concerning the LOS distribution. The parameter λ is calculated based on the user’s choices. If the user enters an LOS, x, and indicates that it is the mean of the distribution, then λ=1/x. If the user enters an LOS and indicates that the entered number is the y^th^ percentile of the LOS distribution, then λ=(1/x)×ln(100/(100-y)). For example, if the user specifies that the value entered is the 75^th^ percentile of the LOS, then λ=ln(4)/x. For example, if the user specified that the mean LOS was 3 days, then we assumed that LOS was exponentially distributed with parameter λ=1/3≈0.333; if the user specified that the 75^th^ percentile of LOS was 5 days, then we assumed that LOS was exponentially distributed with parameter λ=ln(4)/5≈0.277.

Because we assumed that LOS follows an exponential distribution, the probability that a patient exits a ward each day is the same for all patients of a given type (COVID or non-COVID) in each ward each day, regardless of how long they have previously been in the ward. We refer to this value as the “probability of exit”, or Pr(exit). Once we have the parameter λ from the exponential distribution, we calculate the ward-specific probability of departure as Pr(exit) = Pr(LOS < 1 day) = 1 – exp (-λ), where 1 – exp (-λ) is the cumulative distribution function (CDF) of the exponential distribution. In our implementation we adjusted value of Pr(exit) to be between 0.001 and 0.999 to avoid errors from the binomial calculation in the next step. (i.e., calculated values of Pr(exit) below 0.001 were rounded up to 0.001, and values above 0.999 were rounded down to 0.999).

## Simulating the number of Patients that Exit a Ward Each Day

Following the discussion in the previous section, the number of patients discharged from each ward each day follows a binomial distribution where n, the number of trials, is the ward census the previous day, and p, the probability of success, is the probability of discharge per patient per day for that type of ward. In Excel, we simulate the number of exits per patient type using the following formula:

BINOM.INV(number of patients, probability of exit, RAND())

As noted in the previous section, the probability of discharge was truncated because the BINOM.INV function returns error values if Pr(exit) is 0 or 1.

Note that our approach is different than the approach taken by Klein and Reinhardt (2012) [4]. They use the exponential distribution to calculate an LOS value for individual patients, whereas we use the exponential distribution to calculate the probability that exits a ward on any given day. We then use that probability as the probability of success parameter in a binomial distribution where the number of trials, n, is the number of patients in the ward at the beginning of the day.

## Simulation Steps

For each simulated day of the model, the simulation model performs the following steps.

1. Generate the number of new COVID and non-COVID patients in each ward daily. For non-COVID patients, the number of new patient arrivals is simulated using a truncated normal random variable described above. For COVID patients, the number of new arrivals is the sum of the planned intake from other regions and the number of new arrivals from the local region, which is simulated using a truncated normal random variable.

2. For each type of patient, simulate the number of each type of patients to leave a ward each day using a binomial random variable.

3. The number of each patient type at the end of each day is the number of patients at the end of the previous day plus the number of patients who arrive minus the number of patients who exit.

4. Steps 1-3 are repeated to generate an end-of-day census up to the end of the 8^th^ day into the future for each of the six wards.

Since steps 1 and 2 involve random variables, the model is run 400 times to generate a distribution of possible outcomes. The sample size was big enough to produce a wide range of outcomes, allowing the model to execute quickly. A model user can change this number. As described elsewhere, model replications were generated using Excel’s “Data Tables” feature [5]. On a laptop with a 12^th^-generation Intel i7 running at 2.20 GHz, with 16.0 GB of RAM and 64-bit Windows, using Excel 365, with no other applications running, 400 replications takes less than 1 second; extending the number of replications to 1000 takes approximately 1 second; and extending to 10,000 takes approximately 6 seconds.

## References

1. Bell, P.C. and G.S. Zaric, *Analytics for Managers: With Excel*. 1st ed. 2012, New York: Routledge.

2. Hillier, F.S.L., Gerald J, *Introduction to operations research*. 2015, New York, NY: McGraw-Hill. 1010.

3. Ross, S.M., *Introduction to probability models: Ninth edition*. 2007, Amsterdam ; Boston: Academic Press.

4. Klein, M.G. and G. Reinhardt, *Emergency department patient flow simulations using spreadsheets.* Simul Healthc, 2012. **7**(1): p. 40-7.

5. Zaric, G.S., *Monte Carlo Simulation in Excel Without Using Add-ins*. 2022, Ivey Publishing: London, ON.
